# Consensus Guidelines for the Use of Vosoritide in Children with Achondroplasia in Australia

**DOI:** 10.3390/children11070789

**Published:** 2024-06-28

**Authors:** Louise Tofts, Penny Ireland, Tracy Tate, Supriya Raj, Theresa Carroll, Craig F. Munns, Stephen Knipe, Katherine Langdon, Lesley McGregor, Fiona McKenzie, Andreas Zankl, Ravi Savarirayan

**Affiliations:** 1 Health and Human Sciences, Macquarie University, Macquarie Park, Sydney, NSW 2109, Australia; 2 Queensland Children’s Hospital, South Brisbane, QLD 4101, Australia; 3 School of Health and Rehabilitation Sciences, University of Queensland, St Lucia, QLD 4072, Australia; 4 Murdoch Children’s Research Institute, Parkville, VIC 3052, Australia; 5 Kids Rehab, The Children’s Hospital at Westmead, Sydney, NSW 2145, Australia; 6 Child Health Research Centre, University of Queensland, South Brisbane, QLD 4101, Australia; 7 The Newcastle Paediatric Clinic, New Lambton Heights, NSW 2305, Australia; stephen.knipe@health.nsw.gov.au; 8 Perth Children’s Hospital, Perth, WA 6009, Australia; 9 Telethon Kid’s Institute, Perth, WA 6009, Australia; 10 Women’s and Children’s Hospital, Adelaide, SA 5006, Australia; 11 Genetic Health WA, King Edward Memorial Hospital, Perth, WA 6008, Australia; 12 School of Paediatrics and Child Health, University of Western Australia, Perth, WA 6009, Australia; 13 Faculty of Medicine and Health, The University of Sydney, Sydney, NSW 2050, Australia; 14 Garvan Institute of Medical Research, Darlinghurst, NSW 2010, Australia; 15 Department of Clinical Genetics, The Children’s Hospital at Westmead, Sydney, NSW 2145, Australia; 16 Murdoch Children’s Research Institute, Royal Children’s Hospital, University of Melbourne, Parkville, VIC 3052, Australia

**Keywords:** achondroplasia, treatment, clinical guidelines, vosoritide, rare disease, Australia

## Abstract

Background: Achondroplasia, the most prevalent skeletal dysplasia, stems from a functional mutation in the fibroblast growth factor receptor 3 gene, leading to growth impairment. This condition presents multifaceted medical, functional and psychosocial challenges throughout childhood, adolescence and adulthood. Current management strategies aim to minimise medical complications, optimise functional capabilities and provide comprehensive supportive care. Vosoritide (trade name: VOXZOGO^®^, BioMarin Pharmaceuticals) is the first disease-modifying pharmaceutical treatment approved for the management of patients with achondroplasia and became available in Australia in May 2023. Methods: Standardised clinical guidelines for its optimal use are not yet widely available. To address this gap, a multidisciplinary Australian Vosoritide Working Group, comprising 12 experts with experience in achondroplasia management from across Australia, developed recommendations to guide the use of vosoritide in clinical practice. Results: The recommendations, which are expert opinions of the Australian Vosoritide Working Group, aim to (i) standardise the use of vosoritide across Australia, (ii) support the safe clinical rollout of vosoritide and (iii) support universal access. Conclusions: These recommendations have been developed for healthcare professionals and institutions that are engaged in using vosoritide in the management of achondroplasia and will be revised using a formal framework for clinical guideline development once more evidence is available.

## 1. Introduction

Achondroplasia, a rare disease that is caused by an activating mutation in the fibroblast growth factor receptor 3 (*FGFR3*) gene, affects endochondral ossification and disrupts long bone growth, leading to skeletal disproportion with short stature (125 to 134 cm average adult height) [1,2,3,4,5]. Achondroplasia is associated with several medical complications, including spinal cord stenosis and compression (including at the craniocervical junction); spinal and lower limb deformities (lordosis, kyphosis and bowing of legs); respiratory issues (sleep disordered breathing); and ear, nose and throat complications (middle ear infection and upper airway concerns). Current management approaches include medical or surgical interventions to manage complications, and rehabilitation or allied health interventions to reduce the impact of short stature and limited reach, whilst maximising functional capabilities [1,2,3,4,5]. Historically, there have been no therapeutic options that address the underlying pathophysiology or that increase growth velocity in patients with achondroplasia.

Vosoritide, an engineered C-type natriuretic peptide (CNP) analogue, activates B-type natriuretic peptide receptor signalling with the inhibition of *FGFR3* downstream signalling, leading to the promotion of chondrocyte proliferation and differentiation, and subsequent increased endochondral bone formation [6,7,8,9,10,11,12]. It has a longer half-life than endogenous CNP, allowing for daily subcutaneous administration. Vosoritide has demonstrated efficacy in increasing growth in patients with achondroplasia. Phase 3 clinical trials of vosoritide demonstrated an increase in annualised growth velocity of 1.57 cm/year in children with achondroplasia aged 5 years and older, which were sustained over 3 years [6,7,8,9,10,11,12,13]. Long-term follow-up studies of Phase 2 participants, up to 7 years, have demonstrated sustained improvements in height and disproportionate growth with no evidence of accelerated skeletal maturation [9]. The safety profile of vosoritide was found to be acceptable in clinical trials and in post-marketing settings [6,7,8,9,10,11,12,13]. The sustained treatment effects with vosoritide were observed in children aged 2 to 5 years and those >5 years [7,8,9,10,11]. Preliminary trial data in infants aged 4.4 to 59.8 months demonstrated improvements in height and disproportionate growth with vosoritide, although the number of trial participants in the infant age group was low [12]. The foramen magnum grows rapidly during the first few months of life [12,14] and this process is significantly impaired in achondroplasia [13]. Thus, vosoritide may reduce the risk of foramen magnum stenosis if treatment is begun in infancy. The effects of vosoritide on the foramen magnum in infants aged <2 years are currently under investigation [15].

Vosoritide is the first disease-specific, precision medical treatment to be approved for achondroplasia [16]. In Australia, the registration of vosoritide (marketed under the trade name VOXZOGO^®^) by the Therapeutic Goods Administration (TGA) and the subsequent inclusion in the Pharmaceutical Benefits Scheme (PBS) represent landmark advancements in achondroplasia management [17,18]. Initially approved for patients aged 2 years and older, its label was recently extended to encompass use in infants from birth. The current guidelines are the first to provide expert consensus and guidance for the use of vosoritide in patients of all ages with achondroplasia in Australia. These guidelines aim to assist healthcare professionals and health services with the optimal use of vosoritide in clinical practice for patients with achondroplasia. They should be used in addition to the general clinical guidelines for the overall management of the condition [2,4,5].

## 2. Materials and Methods

An Australian Vosoritide Working Group, comprising 12 medical specialists and allied health professionals with representation from most Australian states, was formed in December 2022 to discuss the optimal use of vosoritide in clinical practice. The group included clinical geneticists, paediatricians, physiotherapists, nurse practitioners, paediatric endocrinologists and rehabilitation specialists with extensive experience in the clinical management of achondroplasia. Five participants from two centres had experience in the use of vosoritide in clinical trials. The Australian Vosoritide Working Group representation ensured comprehensive coverage across achondroplasia care and allowed consideration of the challenges in providing comprehensive care across different regions of Australia. A face-to-face meeting of the Australian Vosoritide Working Group members was held in December 2022 to discuss and provide suggestions for the optimal use of vosoritide in achondroplasia service providers and clinics across Australia, including those with limited infrastructure and resources. These suggestions were collated and agreed upon after extensive discussions on the vosoritide clinical evidence collated through literature reviews, clinical knowledge, experience sharing and expert opinion. Following the face-to-face meeting, a series of recommendation statements were developed that were reviewed and revised by the Australian Vosoritide Working Group members using the Within3 platform. A second meeting was conducted virtually in February 2023 to resolve any conflicts and scrutinise the recommendations for content, wording and literature support or collective expert opinion. Consensus was achieved on thirteen recommendation statements. The PBS requirements are included for completeness but were determined by the Pharmaceutical Benefits Advisory Committee (PBAC) and are published on their website.

Since achondroplasia is a rare disease and vosoritide is the first drug to be approved for its management, there were no randomised controlled comparative trials, case-controlled studies, cross-sectional studies, meta-analyses or case reports in the literature to assess the quality of evidence for clinical guidelines. Therefore, the strength and quality of evidence for the recommendations were not assessed based on the Grading of Recommendations Assessment, Development and Evaluation nor the National Health and Medical Research Council’s system and, instead, are based on the expert opinion of the Australian Vosoritide Working Group and published evidence from clinical trials. The strength of the recommendation was determined by evaluating the balance between benefits and risks.

This manuscript covers the PBS eligibility requirements for initiating and continuing vosoritide with reimbursement, and the consensus recommendations for its use. Items are broken down into essential requirements (E) to ensure the safe use of vosoritide in patients with achondroplasia, as determined by a panel of experienced clinicians, and recommended requirements (R) for delivering optimal care with vosoritide use in patients with achondroplasia, when clinically indicated, as determined by a panel of experienced clinicians.

## 3. Prescribing Vosoritide under PBS Authority

The PBS requirements describe the eligibility criteria for initiating and continuing vosoritide with reimbursement as determined by the PBAC (Table 1) [17].

When initiating treatment with vosoritide, the PBS requires that the patient must have a diagnosis of achondroplasia, confirmed by appropriate genetic testing for *FGFR3* mutations that are associated with achondroplasia. Confirmation of an established achondroplasia diagnosis, via genetic testing, is required by both the TGA and PBS [17,18]. Genetic testing must confirm the Gly380Arg mutation in the *FGFR3* gene.

Additionally, patients must not have evidence of growth plate closure as demonstrated by at least one of the following:

1. Bilateral knee X-rays (proximal tibia, distal femur), taken within 6 months of the application if puberty has commenced;
2. Bilateral knee X-rays (proximal tibia, distal femur), taken within 2 years of commencing treatment if puberty has not commenced.
3. An annual growth velocity of greater than 1.5 cm/year as assessed over a period of at least 6 months.

We would expect that the growth velocity criteria would be most frequently used to initiate and continue children on vosoritide. As growth data are required to be obtained over a 6-month period, the X-ray criteria are more practical in infants under 6 months. All height measurements, wherever undertaken, should be taken using regularly calibrated stadiometers (Supplementary Material S1). If a child’s growth slows to less than 1.5 cm/year before late puberty, urgent investigations into the causes of growth failure are required. The typical growth velocity in children with achondroplasia is around 4 cm/year [19,20,21]. Achondroplasia-specific causes of failure to gain linear height include severe progressive kyphoscoliosis and genu varum.

X-rays and dates (the date the treatment commenced and the date of the X-ray) of radiographic evidence must be documented in the patient’s medical records.

In Australia, a unique authority number for each vosoritide prescription must be obtained by an online application with a digital prescriber account to access funding subsidies [22] or by telephone (1800 888 333). The initial dose of vosoritide should be calculated based on patient weight, with reference to the dosing calculator [23], and weight–dose band tables (Supplementary Material S2). To account for weight-related pharmacokinetics, dosing based on the vial strength and injection volume of vosoritide from the table provided in the Vosoritide Product Information is recommended [18].

To continue a patient on vosoritide, the prescriber should confirm that the patient has previously received PBS-subsidised vosoritide for achondroplasia and has no evidence of growth plate closure using either the growth velocity criteria or X-rays, as described previously. Additionally, the need to change dosing based on age, weight and the response of the patient should be assessed before continuing vosoritide, with documentation in the clinical record for the child’s parents and their family doctor. In children below 10 kg of weight, these changes should be assessed in consultation with experts in achondroplasia. The BioMarin medical team should be contacted for advice if needed.

Vosoritide should be stopped if patients show evidence of growth plate closure on an X-ray or have an annual growth velocity of less than 1.5 cm/year in late puberty on two consecutive measures over at least 6 months as they will no longer meet PBS requirements or have potential benefits from continuing.

## 4. Recommendations for the Safe and Effective Use of Vosoritide

The Australian Vosoritide Working Group makes the following recommendations for the optimal use of vosoritide for the clinical management of patients with achondroplasia.

### 4.1. Before Initiating Vosoritide

**Recommendation 1:** Before prescribing vosoritide, the suitability of the patient for vosoritide treatment should be assessed and an achondroplasia diagnosis should be confirmed via genetic testing (Table 1) [17], and documented in the patient’s medical record (E).

A pre-initiation visit should be scheduled with the family and patient to assess the child’s suitability and to provide initial education. The team providing the initial assessment and education may include a paediatric medical subspecialist who is experienced in achondroplasia, nursing staff and allied health professionals. If the patient has an established diagnosis but testing results cannot be located, a new targeted *FGFR3* test for achondroplasia should be obtained. If access to genetics services is limited, it is reasonable to proceed to genetic testing in advance of genetic review. If genetic testing is negative, vosoritide should not be started and the patient should be appropriately referred for further diagnostic work-up. For patients with a new clinical diagnosis of achondroplasia, a referral to a geneticist for confirmation of diagnosis, counselling, education and testing is recommended.

**Recommendation 2:** Patients should be examined during the pre-initiation screening visit to exclude any severe complications of achondroplasia or general health conditions that may impact growth or treatment with vosoritide. Any complications of achondroplasia or other conditions impacting growth should be addressed before starting vosoritide (R).

Patients require medical, radiographic, laboratory and outcome measurement assessments before initiating vosoritide (Table 2). If the patient has severe complications of achondroplasia (craniocervical junction/spinal cord stenosis or compression, spinal deformities, sleep disordered breathing), appropriate medical or surgical management should be undertaken before initiating vosoritide. Infants should undergo a sleep study and an MRI of the craniocervical junction prior to starting vosoritide treatment. Patients should be assessed for any medical condition that could affect growth (e.g., hypothyroidism), and treatment should be optimised before initiating vosoritide. Patients who have cardiac conditions, other conditions predisposing to hypotension or who are taking medications associated with low blood pressure may be at an increased risk of symptomatic hypotension from vosoritide and may require closer monitoring on initial drug delivery. Hypotension is a very common adverse event; however, it is usually asymptomatic and self-resolves [10,18].

**Recommendation 3:** Patients and families should be educated on the potential benefits and side effects of vosoritide, expected outcomes based on age, the need for daily drug administration, adequate storage and refrigeration of vosoritide, regular assessments, follow-up visits, attendance at the training session and the need for long-term monitoring (E).

**Recommendation 4:** Parents, caregivers and patients, where appropriate, should be provided with training on drug administration and injection techniques, including accurate drug reconstitution, regular rotation of injection sites, correct needle positioning, monitoring of any adverse events or injection site reactions and their management (E).

It is recommended that clinicians educate patients on the different aspects of treatment with vosoritide (Supplementary Material S3). This can be undertaken at a screening visit or a first-dose visit and repeated as necessary. Family members, caregivers and/or the child, if appropriate, should be trained in the correct method of drug administration. Injection training would ideally be delivered by a nurse with experience in training families on caregiver-administered subcutaneous injection. If the child wants to self-inject and their treating clinicians approve, the child can also be trained. Patients and families should be informed that the safety profile of the drug has only been established in cases where ≥90% of the prescribed doses are given, and the effects of irregular dosing patterns are unknown. Families should be provided with the Consumer Medicines Information [24]. When initiating vosoritide treatment, families and patients should be informed about expected outcomes and how they may vary with age.

Families should be advised to take the prescription to their local pharmacy and allow a minimum of a week for the pharmacy to order the product, which also applies to repeats. The families should be allowed sufficient time before booking the first-dose visit to allow time to review all the information received and to obtain the medicine from their pharmacy.

**Recommendation 5:** Informed consent from the parent/caregiver and child (if capable of understanding the education) to start therapy with vosoritide should be obtained prior to prescription (E).

### 4.2. Initiating Vosoritide

**Recommendation 6:** For initiating patients on vosoritide, a specialist achondroplasia service is recommended. Patients and families should be supervised during their first-dose administration, and all patients should be monitored afterward to assess for any reactions or adverse events (E).

The first dose of vosoritide should be administered under healthcare supervision to ensure that the techniques used by patients and families are correct and the child tolerates the injection. The patients should be observed for a minimum of 2 h as the half-life of the drug is 28 min. Observation of 1 h for older children (aged 6 years and older) can be considered if there are no complications and the patients are medically stable. Pulse and blood pressure should be checked pre-dose, at 15 and 30 min post-injection, and then every 30 min until the observation period ends. If the child has symptomatic hypotension, a medical review is required. Clinical review, prior to discharge, is recommended to confirm ongoing caregiver administration in the home. Families should be provided with the contact details for their clinical team, emergency contacts for outside of business hours and the local injection support helpline.

**Recommendation 7:** The first follow-up consultation is recommended at 2–4 weeks after the first dose of vosoritide to check for adverse events, injection technique, tolerance, patient compliance, challenges with drug administration and to answer any further questions (R).

The first follow-up consultation could be offered by telephone or video call if this is deemed safe by the treating medical specialist. Families with ongoing concerns about administering vosoritide may benefit from further training or require a face-to-face consultation with a nurse or doctor from the treating team. Clinicians may choose to assess face-to-face at 3 months post-initiation, in line with usual practice for other growth-promoting treatments.

### 4.3. Ongoing Monitoring and Regular Assessments

**Recommendation 8:** Patients who are treated with vosoritide should be assessed regularly to monitor growth, complications of achondroplasia and the efficacy and safety of vosoritide treatment. The ongoing monitoring visits should commence 6 months after the first dose of vosoritide and then should include 6-monthly and annual visits (E).

**Figure 1:**
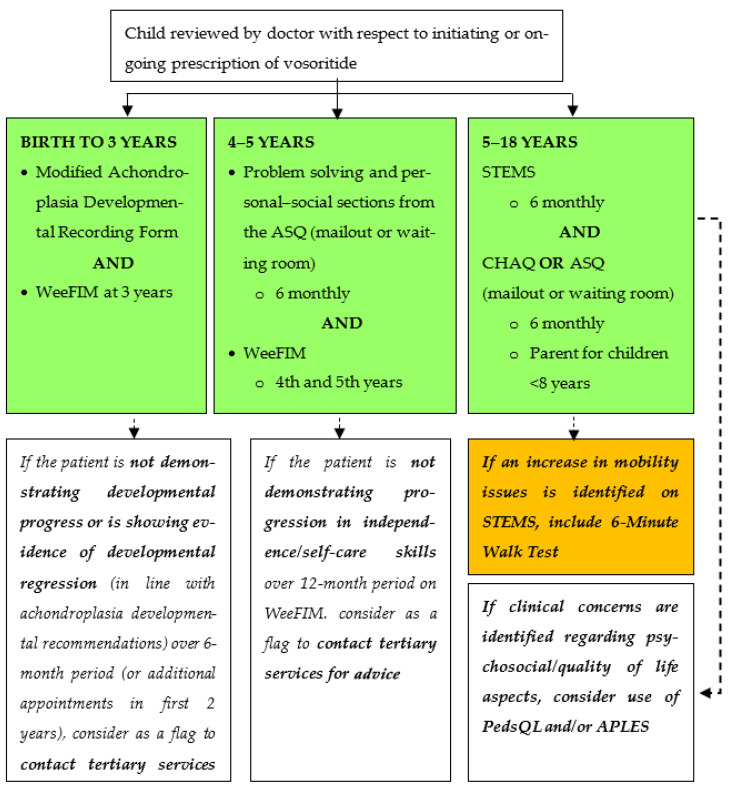
Flowchart for assessing the developmental progress in children with achondroplasia, based on age. APLES, Achondroplasia Personal Life Experience Scale; ASQ, Ages and Stages Questionnaire; CHAQ, Childhood Health Assessment Questionnaire; PedsQL, Paediatric Quality of Life Inventory; STEMS, Screening Tool for Everyday Mobility and Symptoms; WeeFIM, Functional Independence Measure for Children.

Clinical consultations are recommended to monitor the patients after initiating vosoritide treatment and can be combined with routine clinical care appointments. Visits should include essential (E) and recommended (R) assessments to monitor growth-related adverse events of the treatment. Musculoskeletal, neurological or other complications that are associated with achondroplasia should also be assessed (Table 2). Essential assessments are required to ensure the safe use of vosoritide in patients and to help tailor vosoritide treatment in relation to continuation, dose escalation or discontinuation. Recommended assessments provide additional safety assessments and determine the effects of vosoritide on patient function. These assessments can be guided by the clinical picture and available resources. In addition, 6-monthly (interim) visits may be shorter, with longer annual visits including allied health and nursing support to undertake additional assessments. The developmental progress of patients, based on age, should be assessed on a regular basis (Figure 1) using the developmental checklist (Table 3). Anthropometric measurements (Supplementary Material S4) to measure height should be conducted at every visit by using calibrated stadiometers (Supplementary Material S1).

**Recommendation 9:** For patients treated with vosoritide, a specialist achondroplasia clinic is recommended for initiating vosoritide and for ongoing monitoring visits. However, as the population of children with achondroplasia is geographically spread and the PBS requires 6-monthly authority scripts, the option for a ‘shared care’ model between the specialist achondroplasia clinic and their local paediatrician or paediatric endocrinologist may be considered (R).

As per clinical guidelines [2,4,5], patients with achondroplasia require access to subspecialist medical and allied healthcare professionals as part of their routine care. Although a specialist achondroplasia service is recommended for the ongoing monitoring of patients who are treated with vosoritide, it is likely that some children and families will require a ‘shared care’ model between the specialist achondroplasia service and a community paediatric or paediatric subspecialist service, owing to logistical challenges in accessing tertiary health services. When children aged 2 years and older have been established on vosoritide treatment, if the treating achondroplasia specialist considers it safe, they could be co-managed by a general paediatrician for their interim visits. A paediatric endocrinologist with experience in the management of children with growth disorders may also be an option for shared care with the specialist achondroplasia clinic. Some centres have developed an interdisciplinary clinic consisting of a rehabilitation specialist, paediatric endocrinologist, allied health and nursing for optimal care. However, annual visits should be completed in the specialist achondroplasia clinic, which includes specialist nursing and allied health input.

**Recommendation 10:** If any serious adverse events that may be related to the drug are observed, or if the patient is not responding to vosoritide treatment despite having open growth plates, this should be discussed with the specialist clinic for appropriate management (E).

In the case of serious adverse events that are suspected to be related to vosoritide, the patient should be provided with clinical management and a report submitted to the TGA under the Black Triangle Scheme. Identifying worsening spinal deformity and slipped upper capital femoral epiphyses is important as these can be associated with accelerated growth. Current known adverse events are listed in the VOXZOGO^®^ Product Information [18]. Patients who are not responding to vosoritide despite open growth plates should be assessed for conditions like significant scoliosis and knee varus deformities that can result in reduced standing height. Rare unrelated conditions such as growth hormone deficiency may need to be excluded.

### 4.4. Terminating or Suspending Vosoritide

**Recommendation 11:** Treatment should be suspended if the patient has, or is at risk of, low blood pressure; for example, when fluid intake is reduced during severe intercurrent illness and during the perioperative period (suspend until out of recovery) (R).

## 5. Special Considerations for Vosoritide Use in Babies Aged 0 to 24 Months

The Australian Vosoritide Working Group makes the following special recommendations for the safe use of vosoritide in babies aged 0 to 24 months.

**Recommendation 12:** In babies aged <28 days, in addition to the first dose, the second dose of vosoritide should also be administered under medical supervision in a specialist clinic and the observation period post-dose should be 4 h for both the first and the second doses (E).

**Recommendation 13:** In babies and infants < 24 months of age, all monitoring visits (6-monthly and annual) should take place in a specialist clinic (R).

In babies and infants aged 2 years and under, a specialist clinic for initiating vosoritide and ongoing monitoring is recommended to ensure adequate resourcing to conduct the required assessments (Table 2). For these children, a formal review in the specialised clinic should occur 3 months after starting vosoritide. For young children who are unable to stand independently, supine length measurements are needed (Supplementary Material S4). For facilities that are managing children under 24 months of age, a paediatrician in consultation with a medical specialist that is experienced in the management of achondroplasia (PBS requirement), as well as nursing and allied health is highly recommended.

## 6. Conclusions

Vosoritide is the first disease-specific, precision medicine therapy to be approved for use in children with achondroplasia. These recommendations provide a framework to guide healthcare professionals and health services to effectively offer vosoritide as part of a comprehensive management plan for patients with achondroplasia. Due to the novelty of vosoritide, it is expected that there will be logistical and resource-related hurdles across healthcare settings and consideration of diverse patient needs. It is imperative for multidisciplinary teams to remain agile in addressing these challenges while ensuring optimal patient care.

These recommendations should not replace informed clinical judgement that is based on individual patient needs and should be used in conjunction with the broader clinical guidelines [2,4,5] on the overall treatment of achondroplasia as well as the current PBS criteria, TGA registration guidance and prescribing information for vosoritide [17,18]. In the future, as vosoritide use in achondroplasia becomes established and more evidence becomes available, especially for children under 24 months of age, these guidelines will need to be updated using the formal National Health and Medical Research Council grading system. Collaborative networking among specialist achondroplasia clinics and the broader healthcare network across Australia will be instrumental in facilitating knowledge exchange and promoting best practices in this early phase.

These guidelines will serve as a valuable resource for informed decision-making for the use of vosoritide to treat patients with achondroplasia in Australia; however, they should be viewed as a tool to support clinical decision-making that is based on each child’s unique circumstances. Comprehensive information about the known and potential effects, risks and benefits of vosoritide compared with the health risks associated with the condition itself should be provided by clinicians who are experienced in managing achondroplasia to ensure that patients and families can make fully informed decisions about treatment. Over time, the treatment will modify the achondroplasia phenotype, which will be important for families to individually consider. Families should be encouraged to read widely and consider multiple sources of information before initiating therapy. All children with achondroplasia should have equitable access to the best evidence-based care and clinical care as documented in the Australian and International Consensus guidelines, irrespective of their decision regarding vosoritide treatment.

## Figures and Tables

**Table 1:** Summary of requirements for prescribing vosoritide with reimbursement as described by the PBS [17]. This government legislation must be followed by all paediatricians in Australia when using vosoritide.

Treatment Phase	Initial Treatment	Continuing Treatment
Clinical criteria	Patient must have a diagnosis of achondroplasia, confirmed by appropriate genetic testing; AND Patient must not have evidence of growth plate closure, demonstrated by at least one of the following: (i) Bilateral lower extremity X-rays (proximal tibia, distal femur), taken within 6 months of this application if puberty has commenced; (ii) Bilateral lower extremity X-rays (proximal tibia, distal femur), taken within 2 years of commencing treatment if puberty has not commenced; (iii) An annual growth velocity of greater than 1.5 cm/year as assessed over a period of at least 6 months.	Patient must have received PBS-subsidised vosoritide treatment for this condition; AND Patient must not have evidence of growth plate closure, demonstrated by at least one of the following: (i) Bilateral lower extremity X-rays (proximal tibia, distal femur), taken within 6 months of this application if puberty has commenced; (ii) Bilateral lower extremity X-rays (proximal tibia, distal femur), taken within 2 years of commencing treatment if puberty has not commenced; (iii) An annual growth velocity of greater than 1.5 cm/year as assessed over a period of at least 6 months.
Treatment criteria	Must be treated by a medical specialist who is experienced in the management of achondroplasia; OR Must be treated by a paediatrician in consultation with a medical specialist who is experienced in the management of achondroplasia.	Must be treated by a medical specialist who is experienced in the management of achondroplasia; OR Must be treated by a paediatrician in consultation with a medical specialist who is experienced in the management of achondroplasia.
Other criteria	At the time of authority application, medical practitioners must request the appropriate number of vials of appropriate strength(s) to provide sufficient drug (based on the weight of the patient), adequate for 4 weeks, according to the specified dosage in the approved PI. A separate authority prescription form must be completed for each strength requested. Up to a maximum of five repeats will be authorised. Appropriate genetic testing constitutes testing for *FGFR3* gene mutations. In patients where puberty has not commenced, radiographic evidence that epiphyses have not closed must be obtained within 2 years of commencing treatment with vosoritide. X-rays and dates (date commenced treatment and date of X-ray) must be documented in the patient’s medical records. Additional radiographic evidence is not required until the patient has begun puberty. In patients where puberty has commenced, radiographic evidence that epiphyses have not closed must be obtained within 6 months of completing an authority application for vosoritide. X-ray and date taken must be documented in the patient’s medical records.	At the time of authority application, medical practitioners must request the appropriate number of vials of appropriate strength(s) to provide sufficient drug (based on the weight of the patient), adequate for 4 weeks, according to the specified dosage in the approved PI. A separate authority prescription form must be completed for each strength requested. Up to a maximum of five repeats will be authorised. In patients where puberty has not commenced, radiographic evidence that epiphyses have not closed must be obtained within 2 years of commencing treatment with vosoritide. X-rays and dates (date commenced treatment and date of X-ray) must be documented in the patient’s medical records. Additional radiographic evidence is not required until the patient has begun puberty. In patients where puberty has commenced, radiographic evidence that epiphyses have not closed must be obtained within 6 months of completing an authority application for vosoritide. X-ray and date taken must be documented in the patient’s medical records.

PBS, Pharmaceutical Benefits Scheme; PI, prescribing information.

**Table 2:** Schedule of assessments.

	Pre-Initiation Activities	Week 2–4 Follow-Up Visit	6-Monthly Visits	Annual Visits
**Medical assessments**				
Diagnostic genetic testing	PBS			
Patient and family education and consent	E			
Subcutaneous injection education and training	E			
Medical history: new complications, surgery, interventions	E	E	E	E
Adverse events		E	E	E
Concomitant medications	E	R	R	R
Blood pressure and pulse	E		E	E
ECG	R			
Echocardiogram	E ^a^			
Physical examination (especially limbs and spine)	E		E	E
Hip clinical assessment	E		E	E
Tanner Stage of pubertal development (from 10 years in boys and 8 years in girls)	E		E	E
**Radiographic assessments ^b^**				
Bilateral lower extremity X-rays, including proximal tibia and distal femur (within 6 months of each script if pubertal and within 2 years if prepubertal)	PBS			PBS
**Laboratory assessment**				
Thyroid function tests ^c^	R			R ^c^
Renal and hepatic function tests and full blood count with iron studies, calcium magnesium phosphate, parathyroid hormone and immunoglobulin A and antigliadin antibody	R			
**Outcome measures**				
Height ^d^	PBS		PBS	PBS
Calculated growth velocity	PBS		PBS	PBS
Weight	PBS		PBS	PBS
Head circumference until three identical measures are taken	E		E	E
Sitting height	R			R
STEMS	E			E
Achondroplasia-specific developmental checklist for under 3 s ^e^	R			R
Health-related quality of life	R			R
(PedsQL) or APLES				
WeeFIM ^f^	R			R
CHAQ	R			R

^a^ Infants under 28 days only, unless clinically indicated in patients over 28 days of age. ^b^ EOS imaging could be considered for repetitive spinal imaging in large centres. ^c^ Regular thyroid function tests are recommended because thyroid function can independently impact growth; vosoritide is not expected to impact thyroid function. ^d^ Height, sitting height and head circumference measurement guidelines are shown in Supplementary Material S4. ^e^ See Figure 1 and Table 3. ^f^ WeeFIM can only be done by an accredited trained assessor at ages 3, 4 and 5. APLES, Achondroplasia Personal Life Experience Scale; CHAQ, Childhood Health Assessment Questionnaire; E, expert consensus essential requirement; ECG, electrocardiogram; PBS, Pharmaceutical Benefits Scheme requirement for authority; PedsQL, Paediatric Quality of Life Inventory; R, expert consensus recommended requirement if clinically indicated; STEMS, Screening Tool for Everyday Mobility and Symptoms; WeeFIM, Functional Independence Measure for Children.

**Table 3:** Developmental checklist for children with achondroplasia aged 3 years and under.

	Yes	No	Sometimes
0–4 months			
Lifts head when prone			
Reaches for objects			
Smiles			
*Watches face and follows with eyes*			
*Responds to loud noises*			
*Anticipates feeding when sees bottle or breast*			
4–8 months			
Rolls over			
Reverse snow plough ^a^			
Passes object between hands			
Bangs objects together			
*Attempts to get toy out of reach*			
*Giggles/laughs*			
*Turns to sounds*			
*Looks at own hands or objects*			
Babbles			
Purée/smooth solids			
Mashed solids			
8–12 months			
Commando crawls			
Snow plough ^a^			
Bear walking			
Traditional crawling ^a^			
Waves			
Says “mama”			
Anticipates peek-a-boo			
Finger feeding			
Cup drinking			
*Anticipates dressing, and lifts arm/leg to assist*			
12–16 months			
Transitions from lying to sitting			
Transitions from sitting to standing			
Transitions from standing to sitting			
Stands holding on			
Walks holding on/cruising			
Scribbles with crayon			
Builds tower with two blocks			
Shakes head			
Imitates single words/sounds			
*Helps to undress,* e.g., *shoes, socks, hat*			
16–20 months			
Stands unsupported			
Walks independently			
Identifies body parts			
Follows one-step request			
Uses single words			
*Understands simple commands*			
Self-feeds with spoon			
*Copies adult activities,* e.g., *wipes up spills, sweeps, combs hair*			
20–24 months			
Unscrews lid from jar			
Builds tower with eight blocks			
Combines two words			
*(Confirm)* Walks independently			
*Kicks a ball*			
*Pretend play—pretend objects are other things,* e.g., *banana is a phone*			
24–28 months			
Draws circle			
Makes short sentences			
*Climbs or crawls up two steps*			
*Follows simple commands,* e.g., *put things away where they belong*			

The parameters in *italics* represent consensus-based items. ^a^ Not always present.

## Data Availability

No new data were created or analysed in this study. Data sharing is not applicable to this article.

## Supplementary Materials

The following supporting information can be downloaded at: https://www.mdpi.com/article/10.3390/children11070789/s1; Supplementary Material S1. Stadiometer calibrations; Supplementary Material S2. Vosoritide (VOXZOGO^®^) dosage calculator: based on patient weight; Supplementary Material S3. Vosoritide (VOXZOGO^®^) home administration education for families; Supplementary Material S4. Anthropometric measurement guide. For additional support and enhanced usability, please visit https://www.biomarin.com/en-au/hcp/ (accessed on 12 May 2024) to access instructional videos and interactive materials to accompany these guidelines.
