# Supplementary material for: Consensus Guidelines for the Use of Vosoritide in Children with Achondroplasia in Australia
Source: Children (Basel). 2024 Jun 28;11(7):789. doi: 10.3390/children11070789 (PMC11274906; doi:10.3390/children11070789)
Supplement: Supplementary file 1 [file children-11-00789-s001.zip › children-3032123-supplementary.pdf]

children

Supplementary materials

Supplementary material S1: Stadiometer Calibrations

To ensure the accuracy of measurements, regular calibration of the stadiometer and supine length board are essential.

Local protocol or specific manufacturer instructions for your stadiometer should be followed. If none exist, please find a standard method of calibration below. You will need a standard-length rod that has been measured using an ISO-certified ruler, which can be obtained from scientific or engineering suppliers. For the rod, metal is preferable over timber as the latter can expand and shrink with temperature changes. Additionally, most hospitals have biomedical engineering departments responsible for calibrating clinical equipment.

Method:

- Using the stadiometer, measure the standard-length rod in a similar way to how you would measure a patient.
- The rod should be touching the back of the stadiometer, if possible.
  - For supine length boards, lay the standard-length rod in the middle of the device and measure as usual.
- The rod should not be tilted or slanted as this could impact the measurement.
- Lower the paddle gently to the top of the rod.
- A measurement within 0.1 cm of the standard-length rod is acceptable.
  - If the measurement is not within 0.1 cm, follow the manufacturer instructions to calibrate according to the accurate measurement.

Supplementary material S2: Vosoritide (VOXZOGO®) Dosage Calculator – Single Dose Volumes Based on Patient Weight

The volume of vosoritide to be administered at the recommended dose is based on the patient's weight and the vosoritide concentration. For practical reasons and to account for weight-related PK changes, the following dosing is recommended.

Body weight (kg)	VOXZOGO® 0.4 mg diluent (water for injections): 0.5 mL concentration: 0.8 mg/mL	VOXZOGO® 0.56 mg diluent (water for injections): 0.7 mL concentration: 0.8 mg/mL	VOXZOGO® 1.2 mg diluent (water for injections): 0.6 mL concentration: 2 mg/mL
3	0.12 mL		
4	0.15 mL		
5	0.20 mL		
6–7	0.25 mL		
8–11	0.30 mL		
12–16		0.35 mL	
17–21		0.40 mL	
22–32		0.50 mL	
33–43			0.25 mL
44–59			0.30 mL
60–89			0.35 mL
≥90			0.40 mL

Supplementary material S3: Vosoritide (VOXZOGO®) Home Administration Education for Families

Demonstration:

1. Explain each piece of equipment required for reconstitution and administration (vial, DTS syringe, EasyPoint® needle, VanishPoint® syringe, alcohol wipes, sharps container) to parents/caregivers.
   - The vial contains the medication in a powder form. It needs to be mixed with the provided sterile water prior to administration.
   - The sterile water comes in pre-prepared syringes (DTS syringes) with the correct amount of water.
   - An EasyPoint® needle is attached to the DTS syringe to allow the water to be injected into the vial. Wipe the top of the vial with an alcohol wipe first to avoid bacteria and contamination.
   - Once the powder is dissolved, a VanishPoint® syringe is used to draw and administer the dose.
   - A sharps container is used to safely store used needles – these can be acquired and disposed of at most pharmacies.

2. Demonstrate the reconstitution and administration (into the skin pad) of VOXZOGO® (vosoritide) for families slowly, talking through each step.

3. Demonstrate the reconstitution and administration (into the skin pad) a second time, but this time ask parents/caregivers to follow along. Perform each step and allow them to copy, providing feedback on their technique as you go.

4. Provide the family with an opportunity to ask questions throughout and at the completion of the demonstration.

Explanation/Discussion:

Cover the following training points (if not already covered throughout the demonstration):

- The child should have an age-appropriate drink and can also have a light snack around 30 minutes prior to injection – this is to prevent potential side effects that could be caused if a drop in blood pressure occurs.
- If refrigerated, allow the medication to come to room temperature for at least 30 minutes prior to injection. Note: VOXZOGO® can be kept at room temperature (under 30°C) for up to 90 days. Advise families that the medication should be refrigerated if room temperature is above 30°C and not to put unused medication back in the fridge once it is removed.
- Once the water has been added, families have up to 3 hours to administer the dose.
- Injections should be given at roughly the same time every day. The minimum gap between doses is 12 hours.
- Discuss injection sites. Advise caregivers to avoid moles, scars, birthmarks or areas where the skin is tender, bruised, red or hard, and to avoid using the same injection site 2 days in a row (e.g. alternate left/right side even if the child has a preferred body part).
- Teach families to check that the vial is not damaged prior to use and to ensure that the contents are clear and not discoloured or cloudy, and that no particles can be seen after reconstitution.
- Discuss potential serious and less serious side effects and the action to take with each (ensure that families know the emergency number 000, as well as local contact).
- Discuss what to do if the needle does not retract as it should (remove from skin and then retract).
- Provide education regarding not recapping needles.

Other Tips for Families:

- In busy households it is possible that parents may not realise if a dose has already been given. To avoid errors, a calendar to tick off after each day's dose can help ensure the dose is given every day and not given twice by accident. Having a regular routine will also help ensure the injection is given every day (e.g. always given after breakfast or before bed).
- Some children may be nervous about receiving injections, which may be a result of pain. Ways to reduce pain include ice/Buzzy Bee® (more effective in older children) and numbing cream (can be effective in younger children and older children). Note: numbing cream takes approximately 45 minutes to take effect.
- Older children have reported the injections hurt less when they are still. Encourage older children to find a position they are comfortable in to receive the injection and to practice deep breathing or utilise a distraction (e.g. TV or phone) while the injection is administered.
- Younger children may need some help staying still during the injection. It is important that they are in a position they feel safe in. A good way to approach this is to have one person hold the child sitting upright in their lap, hugging their arms and placing their leg over the child's leg to help keep them still while not making them feel vulnerable or overly restrained during the injection.
- Some families have utilised reward systems for young children who struggle with injections, which may be effective.
- Other aspects that may impact the child's experience include the time of day, number of parents/helpers present, distraction, injection site, anticipation (some warning but not too drawn out), discussing injections and their purpose with children, giving the child control when able to (e.g. choosing injection site), involving the child where possible (e.g. put retracted needle in a sharps bin, help get equipment out in advance).

VOXZOGO® (vosoritide) Storage at Home

1. VOXZOGO® (vosoritide) can be kept at room temperature for up to 90 days.
2. If the ambient temperature exceeds 30°C, VOXZOGO® (vosoritide) should be refrigerated in a domestic refrigerator.
3. Once VOXZOGO® (vosoritide) has been taken out of the refrigerator, it should not be put back in.
4. Ensure families are aware of the space needed, especially if they may need to refrigerate the drug.
   - Each box measures 190 (L) x 143 (W) x 119 (H) mm.
   - Each box contains 10 days' worth of VOXZOGO® (vosoritide).

Supplementary material S4: Anthropometric Measurement Guide

Length/Height

Recumbent length for children <2 years old or if unable to stand independently for 1 minute.

Method:

Carer:

- Place patient supine to board.
- Position the head in the Frankfurt horizontal plane (Diagram 1) and cup ears to hold the head in place against the headboard.

Measurer:

- Place hips in alignment and fully extend the legs.
- Hold the feet, slide the foot plate, and apply gentle pressure to the foot plate to flex the feet to plantigrade.

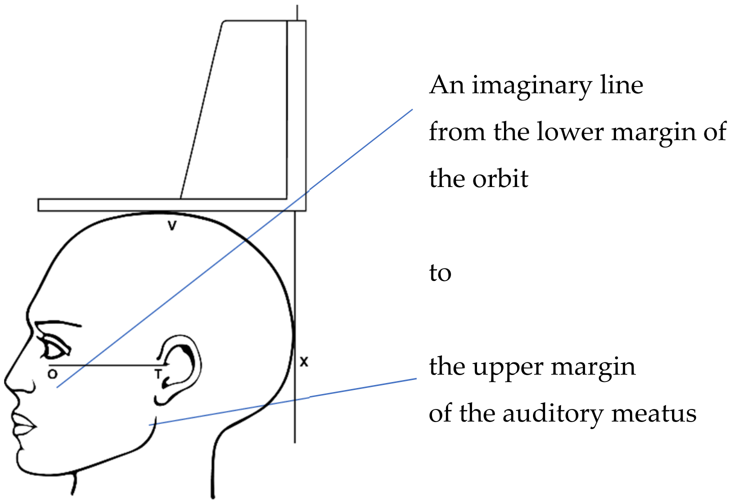

Diagram 1 Frankfurt plane.

Standing Height, Children >2 Years Old

1. All height measurements should be taken with a stadiometer that is regularly calibrated and well maintained.
2. When transitioning from length to standing height, it is best practice to take both measures for the next three visits, as there may be a loss of up to 2 cm in standing height versus length because of gravity.

Method:

- The patient should stand unsupported (make note of scoliosis or contractures).
- Weight is placed evenly on both feet, against the heel plate, and as close together as is comfortable.
- Arms should be relaxed by their sides.
- Heels, bottom, shoulders and head should be touching the back of the stadiometer/wall (but this is not always possible).
- The head should be held with Frankfurt plane parallel to the floor.
- Ask the patient to inhale deeply and maintain a fully erect position. The measurement should be taken at the point of maximum inspiration.

Sitting Height

- The patient should sit on a bench/stool with knees and hips at 90°, hands resting on thighs (feet can be hanging or resting on the floor).
- Buttocks, shoulders and head should be touching the back of the stadiometer/wall.
- The head should be held with Frankfurt plane parallel to the floor.
- Ask the patient to inhale deeply and maintain a fully erect position. The measurement should be taken at the point of maximum inspiration.

Stadiometer measurement – Height of stool = Sitting height

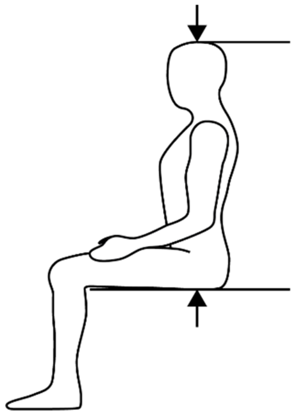

Head Circumference

- Aim for the largest head circumference (occipitofrontal circumference).
- Place the measuring tape just above eyebrow ridges, above the ears and around the back of the head over the occipital prominence.
- Measure twice to ensure accuracy.

Growth Velocity

Standing height (cm) − Previous annual standing height (cm) = Growth velocity (cm/year)

OR

[Standing height (cm) − Standing height (cm)]/Time between measurements (months) × 12 = Growth velocity (cm/year)

Disclaimer/Publisher's Note: The statements, opinions and data contained in all publications are solely those of the individual author(s) and contributor(s) and not of MDPI and/or the editor(s). MDPI and/or the editor(s) disclaim responsibility for any injury to people or property resulting from any ideas, methods, instructions or products referred to in the content.
